# Tangeretin protects renal tubular epithelial cells against experimental cisplatin toxicity 

**DOI:** 10.22038/ijbms.2018.32010.7691

**Published:** 2019-02

**Authors:** Saeed Nazari Soltan Ahmad, Nadereh Rashtchizadeh, Hassan Argani, Leila Roshangar, Amir Ghorbanihaghjo, Davoud Sanajou, Fatemeh Panah, Zahra Ashrafi Jigheh, Siavoush Dastmalchi, Ashkan Kalantary-Charvadeh

**Affiliations:** 1 Department of Biochemistry, Faculty of Medicine, Tabriz University of Medical Sciences, Tabriz, Iran; 2 Student Research Committee, Tabriz University of Medical Sciences, Tabriz, Iran; 3 Biotechnology Research Center, Tabriz University of Medical Sciences, Tabriz, Iran; 4 Urology and Nephrology Research Center, Beheshti University of Medical Sciences, Tehran, Iran; 5 Stem Cell Research Center, Tabriz University of Medical Sciences, Tabriz, Iran

**Keywords:** Cisplatin, Kidney functions, KIM-1, Nephrotoxicity, NGAL, Tangeretin, Tubular injury

## Abstract

**Objective(s):**

Cisplatin is an effective antineoplastic agent; its clinical utility, however, is limited by a few salient toxic side effects like nephrotoxicity. This study aimed to determine the potential protective effects of tangeretin, a citrus-derived flavonoid, against renal tubular cell injury in cisplatin-induced renal toxicity of rats.

**Materials and Methods:**

Tangeretin was injected intraperitoneally at 2.5 and 5 mg/kg doses for 10 days, and a single dose of cisplatin (8 mg/kg) was injected on the 7th day. Tests of kidney function and tubular injury in renal tissues and urine together with oxidative stress and inflammation markers were examined.

**Results:**

Tangeretin ameliorated cisplatin-induced elevations in serum creatinine, BUN, and histopathologic changes. It also attenuated kidney oxidative stress elicited by cisplatin as demonstrated by reduced MDA and increased GSH, CAT, and SOD activities, elevated Nrf2 expression and protein levels of its downstream effectors, HO-1 and NQO-1. Tangeretin further alleviated inflammation evoked by cisplatin as indicated by reduced NF-κB p65 subunit phosphorylation with a simultaneous decrement in its downstream effectors IL-1β and TNF-α expression and protein levels. Moreover, it declined caspase-3 protein levels and TUNEL positive cells in the kidneys, the markers of apoptosis and DNA fragmentation, thus improving renal endurance. Additionally, tangeretin mitigated renal levels of KIM-1 and NGAL, as well as urinary cystatin C and β2-microglobulin concentrations, the markers of renal tubular injury.

**Conclusion:**

Collectively, these data signify the binary profit of tangeretin: enhancement of renal protective mechanisms against cisplatin and attenuation of renal tubular cell injuries induced by the agent.

## Introduction

Cisplatin, a platinum-containing chemotherapeutic agent, is routinely used for the management of various solid organ tumors; however, the charge neutrality and low molecular weight of the drug makes it prone to be freely filtered by the renal glomeruli and subsequent reabsorption and accumulation in the proximal tubular cells; the events that culminate in renal tubular cell damage (1). Therefore, nephrotoxicity is a major issue, limiting the clinical utility of cisplatin (2). The adverse effects of the agent have been related to either long-term (3) or high-dose (2) administrations, both inevitable to some extent in cancer patients.

Cisplatin-induced nephrotoxicity is accompanied by a rise in the levels of reactive oxygen species (ROS) that evoke tissue inflammation with resultant necrosis and apoptosis (4). Though implementing measures to increase urine volume by diuretics and/or heavy hydration have been fairly successful in reducing the risk of renal tubular damage, still, more than 30% of patients experience nephrotoxicity after the first dose of cisplatin (5, 6).

As increased production of inflammatory cytokines such as interleukin-1β (IL-1β) and TNF-α has also been demonstrated after cisplatin administration (7, 8), the suppression of oxidative stress together with inflammation have improved kidney function after cisplatin-induced nephrotoxicity (9-11). It is worth noting that NF-E2-related nuclear factor-2 (Nrf2) and its repressor keap1, regulate the expression of genes coding for antioxidant enzymes such as superoxide dismutase, catalase, heme oxygenase-1 (HO-1) and glutathione peroxidase (GPX) (12, 13); and implementing measures to upregulate the activity of this pathway seems to be an effective way for reducing ROS levels.

Tangeretin, a natural substance present in citrus peels, is highly safe and non-toxic to living cells, and documented to possess conspicuous antioxidant properties (14) with efficient anti-inflammatory effects both *in vitro* and *in vivo* (15-17). Kidney injury molecule-1 (KIM-1) and neutrophil gelatinase-associated lipocalin (NGAL) have emerged as sensitive and specific markers of renal tubular injury, their levels rise at the very early stages of acute kidney injury (18), and have been proposed as accurate urine markers of gentamicin-induced nephrotoxicity in rats (19). Therefore, the aim of the present study was to evaluate the protective effects of tangeretin on renal tubules in rat models of cisplatin-induced nephrotoxicity as assessed by specific markers of tubular damage.

## Materials and Methods

***Animals***

Male Wistar albino rats (8 weeks old) weighing between 180–200 g were acquired from the Animal Care Center, Tabriz University of Medical Sciences. All animal experiments and procedures were conducted with adherence to Guidelines for the Care and Use of Animals in Scientific Research issued by Research Council of the University. Animal Ethics Committee of Tabriz University of Medical Sciences approved the protocol of the study (IR.TBZMED.REC.1395.574). Animals were housed for a week prior to the start of the experiment as an acclimatization period. A controlled temperature (25±1 ^°^C) with relative humidity (65±3%), under a 12-hr dark/light cycle (lights on 7:00-19:00) were the housing conditions throughout the study. During the study period, animals had constant access to standard diet and tap water *ad libitum*.

**Figure 1:**
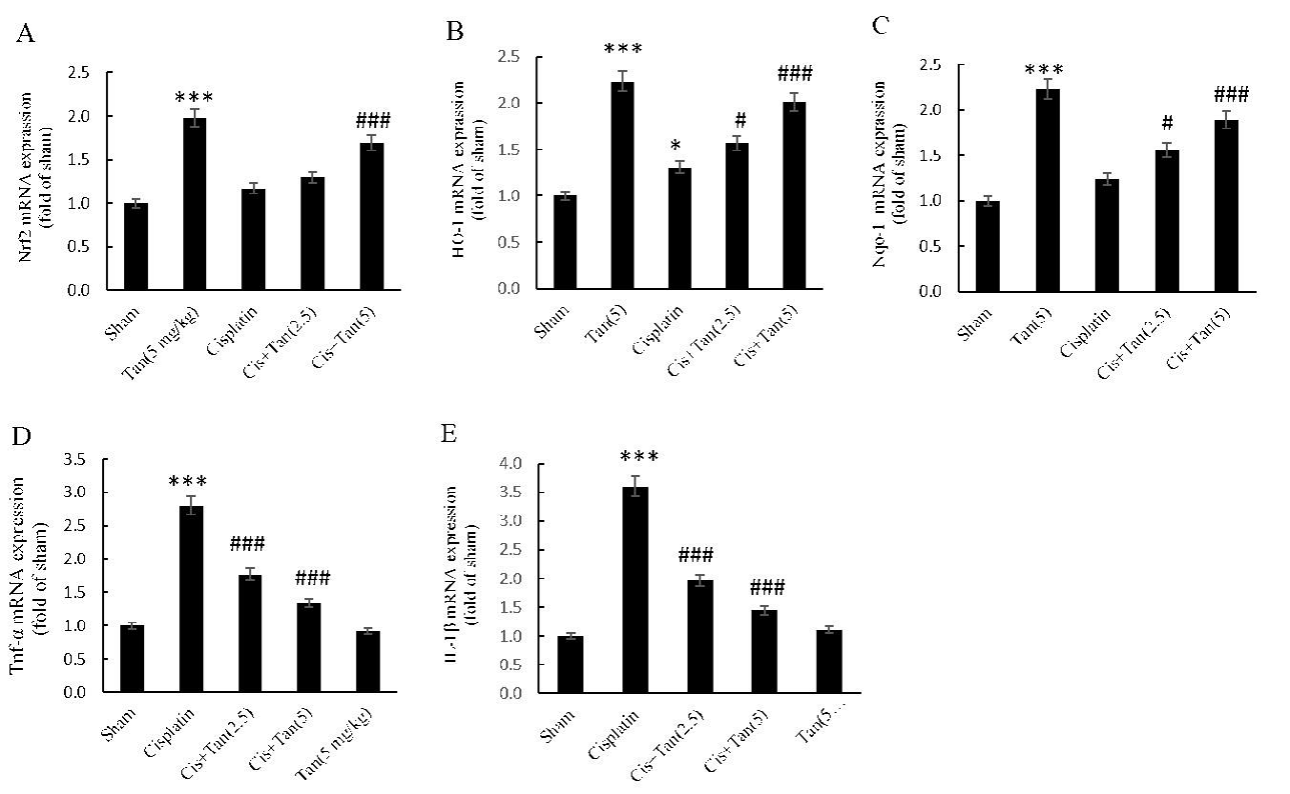
Effects of tangeretin on renal gene expression of Nrf2 (A), HO-1 (B), NQO1 (C), TNF-α (D), and IL-1β (E) as assessed by RT-qPCR. Sham, healthy control rats; Cisplatin, rats treated with 8 mg/kg cisplatin; Cis+Tan (2.5), cisplatin-received rats treated with 2.5 mg/kg tangeretin; Cis +Tan (5), cisplatin-received rats treated with 5 mg/kg tangeretin; Tan (5), healthy rats receiving only 5 mg/kg tangeretin. ***P<0.001 vs sham; #*P<*0.05, ###*P<*0.001 vs cisplatin group

**Figure 2:**
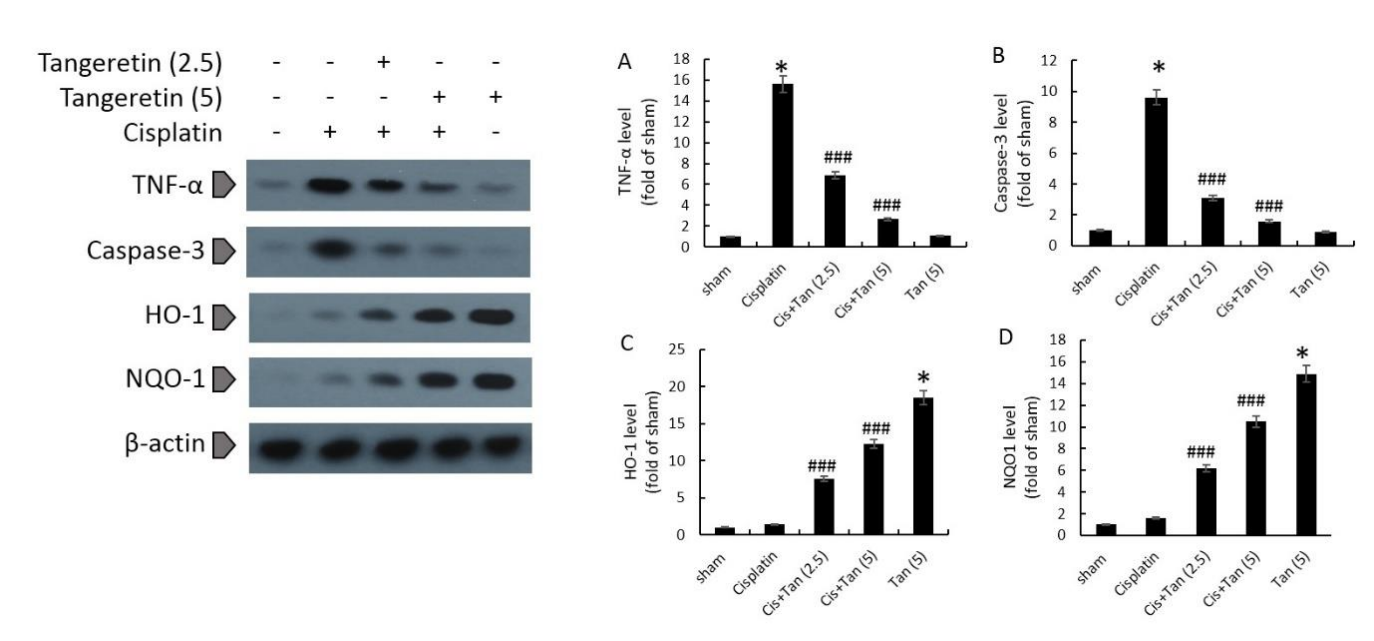
Effects of tangeretin on renal protein levels of TNF-α (A), caspase-3 (B), HO-1 (C), and NQO1 (D) as assessed by Western blotting. Sham, healthy control rats; Cisplatin, rats treated with 8 mg/kg cisplatin; Cis+Tan (2.5), cisplatin-received rats treated with 2.5 mg/kg tangeretin; Cis+Tan (5), cisplatin-received rats treated with 5 mg/kg tangeretin; Tan (5), healthy rats receiving only 5 mg/kg tangeretin. *P<0.05 vs sham; ###*P<*0.001 vs cisplatin group

**Figure 3:**
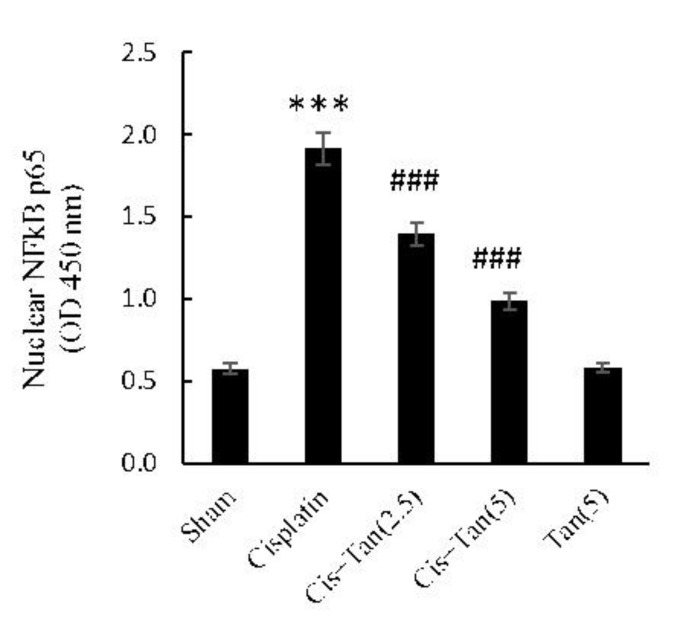
Effect of tangeretin on renal NF-kB p65 activities in nuclear fractions of tissue extracts. Nuclear proteins were extracted from kidney tissue homogenates by using a nuclear extraction kit; the assay was an ELISA-based technique detecting phosphorylated p65 in nuclear extracts; the light absorbance at 450 nm was proportional to the phosphorylated p65 content in micro-wells. Sham, healthy control rats; Cisplatin, rats treated with 8 mg/kg cisplatin; Cis+Tan (2.5), cisplatin-received rats treated with 2.5 mg/kg tangeretin; Cis+Tan (5), cisplatin-received rats treated with 5 mg/kg tangeretin; Tan (5), healthy rats receiving only 5 mg/kg tangeretin. ***P<0.001 vs sham; ###*P<*0.001 vs cisplatin group

**Figure 4:**
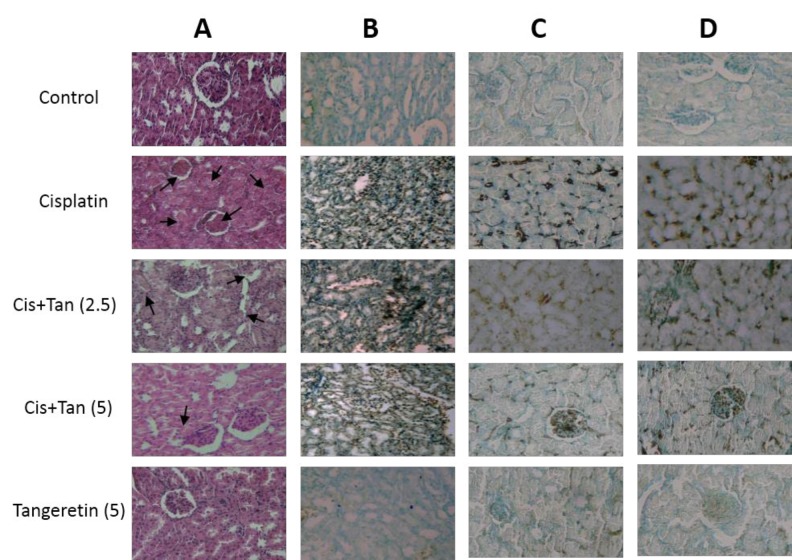
Effects of tangeretin on renal morphological changes (A), renal TUNEL-positive cells (B), renal KIM-1-positive areas (C), and renal NGAL-positive areas (D). A: histopathologic examinations were performed on renal H&E stained sections. C and D: renal immunohistochemical exams. Sham, healthy control rats; Cisplatin, rats treated with 8 mg/kg cisplatin; Cis+Tan (2.5), cisplatin-received rats treated with 2.5 mg/kg tangeretin; Cis+Tan (5), cisplatin-received rats treated with 5 mg/kg tangeretin; Tan (5), healthy rats receiving only 5 mg/kg tangeretin

**Table 1:** HO-1, NQO1, Nrf2, and TNF-α enzymes and GAPDH primers

Gene name	Forward sequence (5'→3')	Reverse sequence (5'→3')
HO-1	ACAGGGTGACAGAAGAGGCTAA	CTGTGAGGGACTCTGGTCTTTG
NQO1	CAGCGGCTCCATGTACT	GACCTGGAAGCCACAGAAG
Nrf2	GGTTATCACAGCTGCCTG	GGTAGTTCTGGGACATGG
TNF-α	GCATGATCCGAGATGTGGAA	GGCTGACTTTCTCCTGGTATG
IL-1β	TACCTATGTCTTGCCCGTGGAG	ATCATCCCACGAGTCACAGAGG
GAPDH	GTCGGTGTGAACGGATTTG	TCCCATTCTCAGCCTTGAC

**Table 2:** Effects of tangeretin on serum urea, serum creatinine, urine cystatin C, and urine β2-MG levels

	Sham	Cisplatin	Cis+Tan (2.5 mg/kg)	Cis+Tan (5 mg/kg)	Tan (5 mg/kg)
Serum urea (mg/dl)	33.5 ± 3.4	95.7 ± 8.6*	71.6 ± 6.33#	59.1 ± 6.18##	33.9 ± 3.9
Serum creatinine (mg/dl)	0.70 ± 0.08	2.10 ± 0.16*	1.23 ± 0.11#	0.98 ± 0.09###	0.67 ± 0.06
Urine cystatin C (μg/g Cr)	47.31 ± 7.64	189.57 ± 32.44*	139.28 ± 42.65#	106.79 ± 36.19##	45.24 ± 8.41
Urine β2-MG (μg/mg Cr)	369.55 ± 15.35	1289.11 ± 67.44*	904.69 ± 88.53#	627.72 ± 93.56##	380.83 ± 28.11

Sham, healthy control rats; Cisplatin, rats treated with 8 mg/kg cisplatin; Cis + Tan (2.5), cisplatin-received rats treated with 2.5 mg/kg tangeretin; Cis + Tan (5), cisplatin-received rats treated with 5 mg/kg tangeretin; Tan (5), healthy rats receiving only 5 mg/kg tangeretin. β2-MG, beta-2 microglobulin; Cr, Urine creatinine.

Values are presented (6 rats per group) as mean ± SD.

* P<0.001 versus sham group;

### P<0.001 vs cisplatin group.

**Table 3:** Effects of tangeretin on renal SOD/CAT activities and GSH/MDA concentrations

	Sham	Cisplatin	Cis+Tan (2.5 mg/kg)	Cis+Tan (5 mg/kg)	Tan (5 mg/kg)
SOD (U/g tissue)	62.5 ± 5.5	30.4 ± 3.4*	39.70 ± 3.2#	52.9 ± 5.8##	65.67 ± 6.2
CAT (U/g tissue)	39.7 ± 3.7	18.8 ± 2.2*	26.3 ± 2.3#	34.3 ± 3.3##	41.3 ± 4.0
GSH (μmol/g tissue)	2.02 ± 2.0	1.09 ± 1.4*	1.62 ± 1.7#	1.88 ± 1.5##	2.14 ± 1.8
MDA (nmol/g tissue)	5.82 ± 0.45	16.52 ± 1.04*	11.36 ± 0.73#	9.58 ± 0.86###	5.78 ± 0.55

Sham, healthy control rats; Cisplatin, rats treated with 8 mg/kg cisplatin; Cis + Tan (2.5), cisplatin-received rats treated with 2.5 mg/kg tangeretin; Cis + Tan (5), cisplatin-received rats treated with 5 mg/kg tangeretin; Tan (5), healthy rats receiving only 5 mg/kg tangeretin. SOD: superoxide dismutase; CAT: catalase; GSH: glutathione; MDA: malondialdehyde.

Values are presented (6 rats in each group) as mean ± SD.

* P<0.001 vs sham group;

# P<0.05,

## P<0.01, and

### P<0.001 vs cisplatin group.

**Figure 5:**
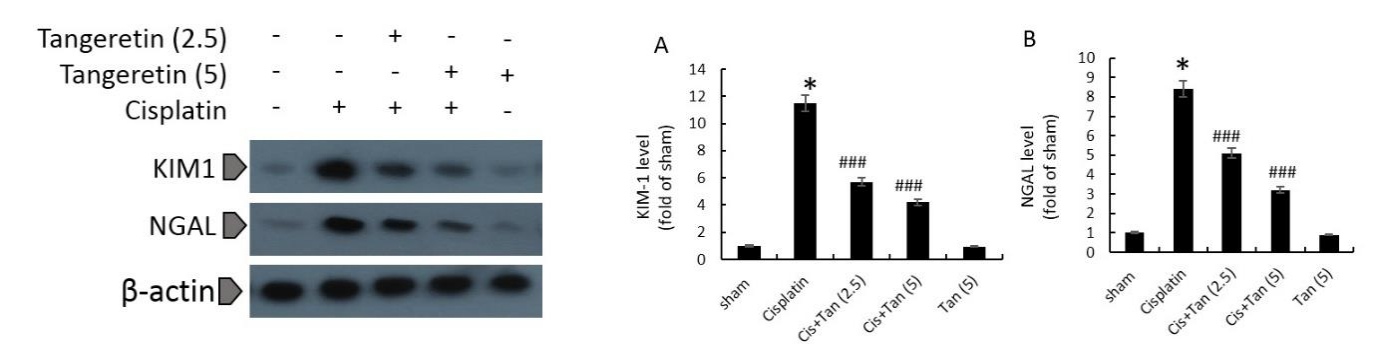
Effects of tangeretin on renal KIM-1 levels (A), and renal NGAL levels (B) as assessed by Western blotting. Sham, healthy control rats;

Cisplatin, rats treated with 8 mg/kg cisplatin; Cis+Tan (2.5), cisplatin-received rats treated with 2.5 mg/kg tangeretin; Cis+Tan (5), cisplatinreceived rats treated with 5 mg/kg tangeretin; Tan (5), healthy rats receiving only 5 mg/kg tangeretin. *P<0.05 vs sham; ###*P<*0.001 vs cisplatin group

***Drugs and chemicals***

Tangeretin and cisplatin were purchased from INDOFINE (Hillsborough, New Jersey, USA) and Sigma–Aldrich (St. Louis, Missouri, USA), respectively. Superoxide dismutase and catalase activity assay kits and also malondialdehyde and total glutathione colorimetric assay kits were obtained from ZellBio (Ulm, Germany). Nuclear protein extraction and NF-κB p65 subunit activity assay kits were purchased from Cayman Chemical (Ann Arbor, Missouri, USA). To perform TUNEL assay, In Situ Cell Death Detection kit (Roche, Manheim, Germany) was utilized. Serum creatinine and BUN levels were determined by Jaffe’s and enzymatic methods, respectively, using commercially available kits (Pars Azmoon, Tehran, Iran). Primary antibodies against NGAL (sc-515876), KIM-1 (sc-518008), NAD (P) H: quinone oxidoreductase-1 (NQO-1, sc-32793), heme oxygenase-1 (HO-1, sc-136960), tumor necrosis factor-α (TNF-α, sc-12744), and caspase-3 (sc-7272) were all obtained from Santa Cruz Biotechnology (Dallas, Texas, USA). Finally, a fluoroimmunoassay (FIA) method was opted for detecting urinary levels of beta-2 microglobulin using commercial kits (BioMérieux, Marcy-l’Étoile, France), and a particle-enhanced nephelometric immunoassay (PENIA) method was adopted to measure urinary cystatin C levels (GoldSite Diagnostics, Shenzhen, China).

**Table 4:** Histopathological assessment of cisplatin-induced damage in rat kidney

	Sham	Cisplatin	Cis+Tan (2.5 mg/kg)	Cis+Tan (5 mg/kg)	Tan (5 mg/kg)
Tubular Damage	0.15 ± 0.04	4.10 ± 0.25^*^	2.08 ± 0.07^##^	1.25 ± 0.04^###^	0.2 ± 0.02
Glomerular injury	0.05 ± 0.03	2.21 ± 0.18 ^*^	1.03 ± 0.12^#^	0.25 ± 0.07 ^###^	0.05 ± 0.02
Inflammatory cells Infiltration	0.06 ± 0.02	3.73 ± 0.54 ^*^	2.1 ± 0.43 ^#^	1.06 ± 0.28 ^##^	0.08 ± 0.03
Vascular damage	0.08 ± 0.03	2.01 ± 0.14 ^*^	1.08 ± 0.06 ^#^	0.25 ± 0.07 ^###^	0.05 ± 0.02
Interstitial damage	0.09 ± 0.04	1.95 ± 0.1 ^*^	0.91 ± 0.24 ^#^	0.26 ± 0.05 ^###^	0.08 ± 0.03
TUNEL-positive cells (%)	6±2	51±17 ^*^	37±19 ^#^	28±7^##^	7±3

Sham, healthy control rats; Cisplatin, rats treated with 8 mg/kg cisplatin; Cis+Tan (2.5), cisplatin-received rats treated with 2.5 mg/kg tangeretin; Cis+Tan (5), cisplatin-received rats treated with 5 mg/kg tangeretin; Tan (5), healthy rats receiving only 5 mg/kg tangeretin. Scores are based on the scale of (0-5), 0 = indicates normal, 1=1-10, 2=11-25, 3=26-45, 4=46-75, and 5=76-100 and presented as mean±SD of 10 fields/slide and six rats per group at the magnification of ×400.

*P<0.001 vs sham group;

#*P<*0.05,

## *P<*0.01, and

###*P<*0.001 vs cisplatin group

***Experimental protocol***

Thirty rats were randomly divided into 5 groups of 6 rats in each. Group 1 (sham group) received intraperitoneal (IP) injections of 0.05% dimethyl sulfoxide (DMSO)-saline (2 ml/kg) for 10 consecutive days. Group 2 (Cis group) received a single IP injection of saline/0.05% DMSO-dissolved cisplatin (8 mg/kg) on the 7th day. Group 3 (Cis+TAN 2.5 group) and group 4 (Cis+TAN 5 group) received IP injections of 0.05% DMSO-dissolved tangeretin (2.5 mg/kg and 5 mg/kg, respectively) for 10 days plus a single IP injection of cisplatin (8 mg/kg) on the 7th day. Group 5 (TAN 5 group) received only IP injections of 0.05% DMSO-dissolved tangeretin (5 mg/kg) for 10 days. Considering a previous pilot study (20), 8 mg/kg IP cisplatin induces a significant nephrotoxicity in rats with the least mortality rate.

Rats were anesthetized by ketamine (75 mg/kg) and xylazine (10 mg/kg) injection (IP) on the 10th day (12 hr after the last treatment with tangeretin). Whole blood was taken by cardiac puncture, centrifuged at 3000 rpm for 15 min to collect serum. Right kidneys were immediately snap frozen in liquid nitrogen and stored at −80 ^°^C for biochemical evaluation and the left kidneys were divided into two sections, fixed in 10% neutral buffered formalin (NBF) for histopathological examinations.

***Assessment of SOD, CAT, MDA, and GHS levels in renal tissues***

Kidney tissues were rinsed with 10% cold phosphate buffered saline (PBS) solution (PH 7.4) to remove any residual blood clot. Tissues were weighed and homogenized in PBS buffer and centrifuged at 8000 rpm for 15 min at 4 ^°^C to collect supernatant fluids; these supernatant fractions were used to measure the desired markers of oxidative stress. Total protein content was measured by the Bradford method, in which Coomassie Brilliant Blue binds to proteins and shifts absorbance from 465 to 595 nm (21).

***Real-time Quantitative PCR assays***

Total RNA mini extraction kit was used for extracting total RNA from kidney tissues. The first-strand cDNA was synthesized according to the manufacturer’s instructions. Amplification of HO-1, NQO1, Nrf2, IL-1β, TNF-α, and GAPDH genes were performed using the following specific primers (Table 1). The SYBR Green Real-Time PCR technique and 2^-ΔΔCT^ formula were applied to assess the relative expression levels of genes in kidney tissues.

***NF-κB p65 subunit activity assay***

One gram of frozen tissue was submerged in liquid nitrogen and ground to obtain a single cell slurry. Nuclear protein was separated using the nuclear extraction kit according to the manufacturer’s instructions. Total protein content was measured in the resultant fraction by the Bradford method and 10 µg protein was loaded per each well. Phosphorylated p65 was captured by consensus dsDNA sequence coated onto the wells and then detected by adding the primary anti-phosphorylated-p65 antibody followed by incubation with HRP-labeled secondary antibody and substrate addition. Each well’s absorbance was read at 450 nm.

***TUNEL assay***

*In situ* TUNEL staining was implemented to detect DNA fragmentation in tissue sections. Briefly, tissue samples were fixed in 10% formalin solution, followed by embedding in paraffin wax. 4-micrometer sections of renal tissues were incubated for 10 min at 37 ^°^C for antigen retrieval. Endogenous peroxidases were inactivated by 3% hydrogen peroxide in methanol for 15 minutes at room temperature. Subsequently, slides were incubated in solutions containing TdT equilibration buffer (10 min at RT) and TdT reaction buffer (30 min at 37 ^°^C). To block nonspecific binding, incubation with BSA solution (40 min at RT) was performed. Final staining was done using the DAB solution.

***Histopathologic assessment***

All tissues were fixed in 10% formalin solution and embedded in paraffin wax according to routine procedures. Sections (4 µm thick) were prepared using a microtome (Leica, Germany) and stained with Hematoxyline and Eosin (H&E). Renal structural abnormalities including tubular necrosis and dilatation, interstitial edema, cast formation, and brush border loss were examined in 20 random fields under a magnification of ×400 and scored based on a scale of 0–5. 0= indicates normal, 1= 1-10, 2= 11-25, 3= 26-45, 4= 46-75, and 5= 76-100. Slides were scored in a blinded fashion and values were expressed as mean±SD.

For immunohistochemical analysis, we followed the same initial procedures described for TUNEL assay, except for incubating slides with primary antibodies after inactivation of endogenous peroxidases. After incubation with an HRP-conjugate, DAB chromogen was used to satin desired foci, and slides were counterstained with toluidine blue.

***Western blotting analysis***

Kidney cortices were homogenized in RIPA buffer solution (Santa Cruz Biotechnology) containing proteinase and phosphatase inhibitors. The concentration of supernatant protein was measured using the Bradford method. The samples were electrophoresed on sodium dodecyl sulfate-polyacrylamide gel electrophoresis (7–14%) gels, followed by the transfer to polyvinylidene difluoride membranes. The blots were incubated with primary and secondary antibodies and visualized using Pierce ECL Western Blotting Substrate (Thermo Fisher Scientific, Waltham, Massachusetts, USA).

***Statistical analysis***

Statistical analysis was performed using SPSS software (version 16.0, SPSS). Values were expressed as mean±SD. One way analysis of variance (ANOVA) was used followed by Tukey-Kramer multiple comparisons tests to determine statistical significance difference between control and multiple experimental groups. *P*<0.05 was considered significant.

## Results

***Serum creatinine and BUN levels***

As shown in Table 2, a significant increase was observed in serum creatinine and BUN levels after cisplatin administration, and both 2.5 mg/kg and 5 mg/kg pretreatments of tangeretin significantly decreased serum creatinine and BUN levels (Table 2). 

***Renal SOD and CAT activities, and MDA and GSH levels***

As demonstrated in Table 3, a significant decline in SOD and CAT activities and GSH levels together with a significant rise in MDA concentrations in kidney tissues were observed in the cisplatin group. Though pretreatment with 2.5 mg/kg tangeretin increased the activities of SOD and CAT and concentrations of GSH in kidney tissues compared with the cisplatin group, these effects turned out to be more pronounced with 5 mg/kg tangeretin pretreatment and more substantial increases in SOD and CAT activities and GSH levels were noted in these rats, indicating that tangeretin acted in a dose-dependent manner (Table 3).

***Renal expression of HO-1, NQO-1, Nrf2, IL-1β, and TNF-α genes***

Tangeretin alone in normal rats greatly increased the renal expression of all three Nrf2, HO-1, and NQO-1 genes. Moreover, cisplatin itself significantly elevated the expression of HO-1 gene. While 2.5 mg/kg tangeretin pretreatment had no effect on the renal expression of the Nrf2 gene, 5 mg/kg pretreatment elevated its expression as compared to the cisplatin group (Figure 1). The expression of HO-1 and NQO-1 genes, on the other hand, demonstrated dose-dependent increases with 2.5 mg/kg and 5 mg/kg tangeretin pretreatments (Figure 1). The expression of TNFα and IL-1β genes elevated significantly in the cisplatin group and once again a dose-dependent decline in their expressions was seen after 2.5 mg/kg and 5 mg/ kg tangeretin pretreatments in comparison with the cisplatin group (Figure 1).

***Renal levels of HO-1, NQO-1, TNF-α, and caspase-3 proteins***

Tangeretin alone increased the protein expression of HO-1 and NQO-1, however, it had no effect on the expression of TNF-α and caspase-3 proteins (Figure 2). While cisplatin increased slightly the protein levels of HO-1 and NQO-1, it significantly up-regulated TNF-α and caspase-3 protein levels in the kidneys (Figure 2). 2.5 mg/kg and 5 mg/kg tangeretin pretreatments increased further the protein levels of HO-1 and NQO-1 dose-dependently, and meanwhile decremented TNF-α and caspase-3 protein levels in renal tissues (Figure 2).

***NF-κB p65 subunit phosphorylation***

As depicted in Figure 3, NF-κB p65 subunit phosphorylation increased significantly in the cisplatin group as compared to shams. Tangeretin pretreatment dose-dependently suppressed the phosphorylation of NF-κB p65 subunit as observed after 2.5 mg/kg and 5 mg/kg pretreatments.

***Renal histological parameters***

The kidney sections from sham and tangeretin groups revealed normal renal appearances in cortical regions. In contrast, those from cisplatin-treated rats demonstrated extremely severe tubular and interstitial alterations accompanied by an infiltration of the inflammatory cells into the perivascular and subvascular areas. 2.5 mg/kg and 5 mg/kg pretreatments of tangeretin, however, markedly improved histological changes induced by cisplatin (**Table 4 **and Figure 4A).

***DNA fragmentation rates in renal tissues***

TUNEL stained sections from the cisplatin group had a significantly increased number *of* *TUN*EL positive cells. However, a significant decline was noticed in the numbers of these cells after 2.5 mg/kg and 5 mg/kg pretreatments of tangeretin, which again revealed to be dose-dependent in nature (Figure 4B).

***Kidney tissue and urinary markers of renal tubular injury***

As revealed by immunohistochemical and blotting analyses of renal tissue, rats in the cisplatin group had highly elevated levels of renal KIM-1 and NGAL (Figures 4C, 4D, and 5). Furthermore, high urinary levels of cystatin C and *β*2-microglobulin were noted in cisplatin rats as compared to normal ones (Table 2). 2.5 mg/kg pretreatment with tangeretin reduced slightly albeit significantly the levels of renal NGAL and KIM-1 (Figures 4C, 4D, and 5). Urinary cystatin C and *β*2-microglobulin levels were similarly decreased in these rats (Table 2). The most significant reductions of renal KIM-1 and NGAL along with urinary cystatin C and *β*2-microglobulin levels were observed in the group receiving 5 mg/kg tangeretin (Table 2, Figures 4C, 4D, and 5).

## Discussion

The current study showed that tangeretin, an active flavonoid of citrus peels, protected the kidneys against oxidative stress and inflammations induced by cisplatin. In addition to improving renal function as demonstrated by reduced serum levels of BUN and creatinine, tangeretin successfully declined renal and urinary levels of tubular injury markers in cisplatin-induced nephrotoxicity. 

Cisplatin nephrotoxicity is a common side effect, developing in 20% to 30% of patients that receive the drug (22). It is now known that cisplatin-induced oxidative stress increases translocation of Nrf2 into the nucleus; phosphorylated Nrf2 is capable of binding to antioxidant response element (ARE), up-regulating ARE-associated gene expression. Therefore, the levels of key enzymes that augment cellular defense against oxidative stress are elevated including HO-1, NQO1, and glutathione S-transferase (GST) (23). Increased levels of HO-1 and NQO1, apart from promoting antioxidant properties, exert anti-inflammatory and anti-apoptotic effects (24). Thus, devising novel measures to amplify this protective antioxidant system are highly warranted in patients receiving cisplatin.

To date, a number of different chemical agents has successfully been studied in experimental models of cisplatin nephrotoxicity in an attempt to promote antioxidant defense mechanisms and to suppress kidney inflammation (9, 25, 26). Recently, it has been shown that tangeretin reduces renal MDA and nitric oxide (NO) levels and concomitantly increases GSH concentrations and GPX activity in cisplatin-induced nephrotoxic rats; at the same time, renal levels of TNF-α decreased and IL-10 was elevated (27), which resulted in reduced cell apoptosis as indicated by a decline in caspase-3 levels in the kidneys (27). In agreement with these findings, we showed that renal activities of catalase and superoxide dismutase were decreased, and MDA and GSH levels were increased. Furthermore, a significant decline in TNF-α and IL-1*β* gene expressions and TNF-α protein levels in kidney tissue were noted. Caspase-3 protein levels and TUNNEL-positive cells were similarly attenuated in the renal tissues. While vitamin C, vitamin E, and selenium supplementation in cancer patients receiving cisplatin failed to improve serum levels of antioxidant markers (e.g. MDA) (28), devising trials to assess citrus-originated tangeretin effects clinically seems to be plausible, based on our findings.

We know that renal KIM-1 and urinary clusterin levels increase early in the course of cisplatin-induced nephrotoxicity in rats as indicators of tubular cell injuries (29). It has been proposed that daidzein, an antioxidant isoflavone, ameliorates renal tubular cell injuries in rat models of cisplatin nephrotoxicity by demonstrating decreased levels of NGAL in renal tissues and reduced urinary levels of KIM-1 (8). While it is known that tangeretin alleviates renal inflammation and oxidative stress in cisplatin-induced nephrotoxic rats (27), its effects on the markers of tubular injury remain to be elucidated. Therefore, we assessed renal KIM-1 and NGAL levels together with urinary levels of cystatin C and *β*2-microglobulin. We found that tangeretin dose-dependently reduces renal KIM-1/NGAL and urinary cystatin C/*β*2-microglobulin levels in cisplatin nephrotoxicity of rats. Furthermore, serum levels of BUN and creatinine turned out to be declined in a dose-dependent manner by tangeretin, which reflects amelioration in the kidney functions.

As stated previously, cisplatin-associated oxidative stress induces the Nrf2/ARE signaling pathway (23), and it has been demonstrated that tangeretin reduces NF-κB p65 subunit protein levels and Nrf2 activity as evaluated by immunohistochemistry and ELISA, respectively, in rat models of cisplatin nephrotoxicity (27). Our investigation revealed that tangeretin not only decreases NF-κB p65 subunit activity and Nrf2 expression as evaluated by ELISA and RT-qPCR, respectively, but also reduces kidney HO-1 and NQO-1 gene expressions and protein levels, the two notable antioxidant proteins related to Nrf2/ARE signaling activation. Again, these findings support the anti-inflammatory and antioxidant properties of tangeretin in cisplatin-induced nephrotoxicity of rats.

## Conclusion

The present investigation highlights the efficacy of tangeretin in suppressing renal inflammation along with promoting antioxidant defense in cisplatin nephrotoxic rat models, leading to decreased levels of apoptosis markers (i.e. Caspase-3 and DNA fragmentation) in kidneys, and diminution of renal and urinary markers of tubular cell injury (i.e. KIM-1, NGAL, cystatin C, and *β*-2 microglobulin), as well as improvement in kidney functions.
